# A Three-Threshold Learning Rule Approaches the Maximal Capacity of Recurrent Neural Networks

**DOI:** 10.1371/journal.pcbi.1004439

**Published:** 2015-08-20

**Authors:** Alireza Alemi, Carlo Baldassi, Nicolas Brunel, Riccardo Zecchina

**Affiliations:** 1 Human Genetics Foundation (HuGeF), Turin, Italy; 2 DISAT, Politecnico di Torino, Turin, Italy; 3 Departments of Statistics and Neurobiology, University of Chicago, Chicago, Illinois, United States of America; University College London, UNITED KINGDOM

## Abstract

Understanding the theoretical foundations of how memories are encoded and retrieved in neural populations is a central challenge in neuroscience. A popular theoretical scenario for modeling memory function is the attractor neural network scenario, whose prototype is the Hopfield model. The model simplicity and the locality of the synaptic update rules come at the cost of a poor storage capacity, compared with the capacity achieved with perceptron learning algorithms. Here, by transforming the perceptron learning rule, we present an online learning rule for a recurrent neural network that achieves near-maximal storage capacity without an explicit supervisory error signal, relying only upon locally accessible information. The fully-connected network consists of excitatory binary neurons with plastic recurrent connections and non-plastic inhibitory feedback stabilizing the network dynamics; the memory patterns to be memorized are presented online as strong afferent currents, producing a bimodal distribution for the neuron synaptic inputs. Synapses corresponding to active inputs are modified as a function of the value of the local fields with respect to three thresholds. Above the highest threshold, and below the lowest threshold, no plasticity occurs. In between these two thresholds, potentiation/depression occurs when the local field is above/below an intermediate threshold. We simulated and analyzed a network of binary neurons implementing this rule and measured its storage capacity for different sizes of the basins of attraction. The storage capacity obtained through numerical simulations is shown to be close to the value predicted by analytical calculations. We also measured the dependence of capacity on the strength of external inputs. Finally, we quantified the statistics of the resulting synaptic connectivity matrix, and found that both the fraction of zero weight synapses and the degree of symmetry of the weight matrix increase with the number of stored patterns.

## Author Summary

Recurrent neural networks have been shown to be able to store memory patterns as fixed point attractors of the dynamics of the network. The prototypical learning rule for storing memories in attractor neural networks is Hebbian learning, which can store up to 0.138*N* uncorrelated patterns in a recurrent network of *N* neurons. This is very far from the maximal capacity 2*N*, which can be achieved by supervised rules, e.g. by the perceptron learning rule. However, these rules are problematic for neurons in the neocortex or the hippocampus, since they rely on the computation of a supervisory error signal for each neuron of the network. We show here that the total synaptic input received by a neuron during the presentation of a sufficiently strong stimulus contains implicit information about the error, which can be extracted by setting three thresholds on the total input, defining depression and potentiation regions. The resulting learning rule implements basic biological constraints, and our simulations show that a network implementing it gets very close to the maximal capacity, both in the dense and sparse regimes, across all values of storage robustness. The rule predicts that when the total synaptic inputs goes beyond a threshold, no potentiation should occur.

## Introduction

One of the fundamental challenges in neuroscience is to understand how we store and retrieve memories for a long period of time. Such long-term memory is fundamental for a variety of our cognitive functions. A popular theoretical framework for storing and retrieving memories in recurrent neural networks is the attractor network model framework [1–3]. Attractors, i.e. stable states of the dynamics of a recurrent network, are set by modification of synaptic efficacies in a recurrent network. Synaptic plasticity rules specify how the efficacy of a synapse is affected by pre- and post-synaptic neural activity. In particular, Hebbian synaptic plasticity rules lead to long-term potentiation (LTP) for correlated pre- and post-synaptic activities, and long-term depression (LTD) for anticorrelated activities. These learning rules build excitatory feedback loops in the synaptic connectivity, resulting in the emergence of attractors that are correlated with the patterns of activity that were imposed on the network through external inputs. Once a set of patterns become attractors of a network (in other words when the network “learns” the patterns), upon a brief initial activation of a subpopulation of neurons, the network state evolves towards the learned stable state (the network “retrieves” a past stored memory), and remains in that state after removal of the external inputs (and hence maintains the information in short-term memory). The set of initial network states leading to a memorized state is called the *basin of attraction*, whose size determines how robust a memory is. The attractor neural network scenario was originally explored in networks of binary neurons [1, 2], and then extended from the 90s to networks of spiking neurons [4–7].

Experimental evidence in different areas of the brain, including inferotemporal cortex [8–11] and prefrontal cortex [12–14], has provided support for the attractor neural network framework, using electrophysiological recordings in awake monkeys performing delayed response tasks. In such experiments, the monkey has to maintain information in short-term (working) memory in a ‘delay period’ to be able to perform the task. Consistent with the attractor network scenario, some neurons exhibit selective persistent activity during the delay period. This persistent activity of ensembles of cortical neurons has thus been hypothesized to form the basis of the working memory of stimuli shown in these tasks.

One of the most studied properties of attractor neural network as a model of memory is its storage capacity, i.e. how many random patterns can be learned in a recurrent network of *N* neurons in the large *N* limit. Storage capacity depends both on the network architecture and on the synaptic learning rule. In many models, the storage capacity scales with *N*. In particular, the Hopfield network [1] that uses a Hebbian learning rule has a storage capacity of 0.138*N* in the limit of *N* → ∞ [15]. Later studies showed how the capacity depends on the connection probability in a randomly connected network [16, 17] and on the coding level (fraction of active neurons in a pattern) [18, 19]. A natural question is, what is the maximal capacity of a given network architecture, over all possible learning rules? This question was answered by Elizabeth Gardner, who showed that the capacity of fully connected networks of binary neurons with dense patterns scales as 2*N* [20], a storage capacity which is much larger than the one of the Hopfield model. The next question is what learning rules are able to saturate the Gardner bound? A simple learning rule that is guaranteed to achieve this bound is the perceptron learning rule (PLR) [21] applied to each neuron independently. However, unlike the rule used in the Hopfield model, the perceptron learning rule is a supervised rule that needs an explicit “error signal” in order to achieve the Gardner bound. While such an error signal might be available in the cerebellum [22–24], it is unclear how error signals targeting individual neurons might be implemented in cortical excitatory synapses. Therefore, it remains unclear whether and how networks with realistic learning rules might approach the Gardner bound.

The goal of the present paper is to propose a learning rule whose capacity approaches the maximal capacity of recurrent neural networks by transforming the original perceptron learning rule such that the new rule does not explicitly use an error signal. The perceptron learning rule modifies the synaptic weights by comparing the desired output with the actual output to obtain an error signal, subsequently changing the weights in the opposite direction of the error signal. We argue that the total synaptic inputs (‘local fields’) received by a neuron during the presentation of a stimulus contain some information about the current error (i.e. whether the neuron will end up in the right state after the stimulus is removed). We use this insight to build a field dependent learning rule that contains three thresholds separating no plasticity, LTP and LTD regions. This rule implements basic biological constraints: (a) it uses only information local to the synapse; (b) the new patterns can be learned incrementally, i.e. it is an online rule; (c) it does not need an explicit error signal; (d) synapses obey Dale’s principle, i.e. excitatory synapses are not allowed to have negative weights. We studied the capacity and the size of the basins of attraction for a binary recurrent neural network in which excitatory synapses are endowed with this rule, while a global inhibition term controls the global activity level. We investigated how the strength of external fields and the presence of correlations in the inputs affect the memory capacity. Finally, we investigated the statistical properties of the connectivity matrix (distribution of synaptic weights, degree of symmetry).

## Results

### The network

**Fig 1:**
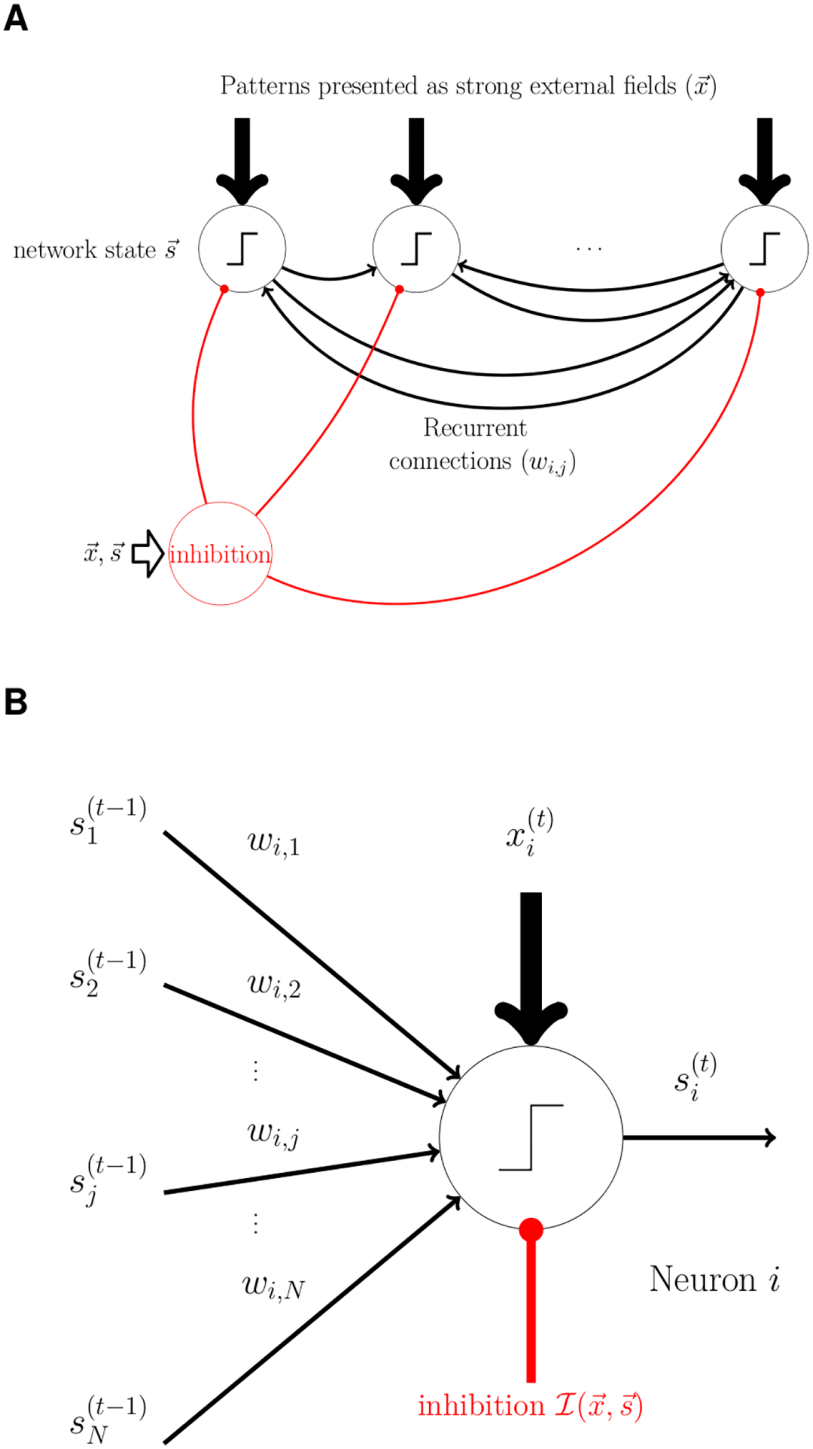
A sketch of the network and the neuron model. **A.** Structure of the network. The fully-connected network consists of *N* binary (*s*_*i*_ ∈ {0,1}) neurons and an aggregated inhibitory unit. The global inhibition is a function of the state of the network and the external fields, i.e. $I(\vec{x},\;\vec{s})$. A memory pattern $\vec{\xi }$ is encoded as strong external fields, i.e. $\vec{x}\;=\;X\;\vec{\xi }$ and presented to the network during the learning phase. **B.** Each neuron receives excitatory recurrent inputs (thin black arrows) from the other neurons, a global inhibitory input (red connections), and a strong binary external field (*x*_*i*_ ∈ {0, *X*}; thick black arrows). All these inputs are summed to obtain the total field, which is then compared to a neuronal threshold *θ*; the output of the neuron is a step function of the result.

We simulated a network of *N* binary (McCulloch-Pitts) neurons, fully-connected with excitatory synapses (Fig 1A). All the neurons feed a population of inhibitory neurons which is modeled as a single aggregated inhibitory unit. This state-dependent global inhibition projects back onto all the neurons, stabilizing the network and controlling its activity level. At each time step, the activity (or the state) of neuron *i* (*i* = 1…*N*) is described by a binary variable *s*_*i*_ ∈ {0,1}. The state is a step function of the *local field* *v*_*i*_ of the neuron:

$$s_{i}=\Theta (v_{i}-\theta ),$$ (1)

where Θ is the Heaviside function (Θ(*x*) = 1 if *x* > 0 and 0 otherwise) and *θ* is a neuronal threshold. The local field *v*_*i*_ represents the overall input received by the neuron from its excitatory and inhibitory connections (Fig 1B). The excitatory connections are of two kinds: recurrent connections from within the excitatory population, and external inputs.

The recurrent excitatory connections are mediated by synaptic weights, denoted by a matrix *W* whose elements *w*_*ij*_ (the weight of the synapse from neuron *j* to *i*) are continuous non-negative variables (*w*_*ij*_ ∈ [0,∞); *w*_*ii*_ = 0). In the following, and in all our simulations, we assume that the weights are initialized randomly before the training takes place (see Materials and Methods).

Therefore, in the absence of external inputs, the local field of each neuron *i* is given by:

$$v_{i}=\sum_{j=1}^{N}w_{ij}s_{j}-I_{0}(\vec{s}),$$ (2)

where $I_{0}\;(\vec{s})$ represents the inhibitory input.

For the sake of simplicity, we simulated a synchronous update process, in which the activity of each neuron *s*_*i*_ is computed from the local field *v*_*i*_ at the previous time step, and all updates happen in parallel.

The network was designed so that, in absence of external input and prior to the training process, it should spontaneously stabilize itself to some fixed overall average activity level *f* (fraction of active neurons, or sparseness), regardless of the initial conditions. In particular, we aimed at avoiding trivial attractors (the all-off and all-on states). To this end, we model the inhibitory feedback (in absence of external inputs) as a linear function of the overall excitatory activity:

$$I_{0}\left(\vec{s}\right)=H_{0}+\lambda (\sum_{i=1}^{N}s_{i}-fN).$$ (3)

The parameters *H*_0_ and *λ* can be understood as follows: *H*_0_ is the average inhibitory activity when the excitatory network has the desired activity level *f*, i.e. when $\sum_{i=1}^{N}s_{i}=fN$; *λ* measures the strength of the inhibitory feedback onto the excitatory network. This expression can be interpreted as a first-order approximation of the inhibitory activity as a function of the excitatory activity around some reference value *fN*, which is reasonable under the assumption that the deviations from *fN* are small enough. Indeed, by properly setting these two parameters in relation to the other network parameters (such as *θ* and the average connection strength) it is possible to achieve the desired goal of a self-stabilizing network.

In the training process, the network is presented a set of *p* patterns in the form of strong external inputs, representing the memories which need to be stored. We denote the patterns as $\left\{\vec{\xi^{\mu }}\right\}$ (where *μ* = 1…*p* and $\xi_{i}^{\mu }\in \{0,1\}$), and assume that each entry $\xi_{i}^{\mu }$ is drawn randomly and independently. For simplicity, the coding level *f* for the patterns was set equal to the spontaneous activity level of the network, i.e. $\xi_{i}^{\mu }=1$ with probability *f*, 0 otherwise. During the presentation of a pattern *μ*, each neuron *i* receives an external binary input $x_{i}=X\xi_{i}^{\mu }$, where *X* denotes the strength of the external inputs, which we parameterized as $X=\gamma \sqrt{N}$. In addition, the external input also affects the inhibitory part of the network, eliciting a response which indirectly downregulates the excitatory neurons. We model this effect as an additional term *H*_1_ in the expression for the inhibitory term (Eq 3), which therefore becomes:

$$I\left(\vec{x},\vec{s}\right)=H_{0}+H_{1}\frac{\sum_{i=1}^{N}x_{i}}{fNX}+\lambda \left(\sum_{i=1}^{N}s_{i}-fN\right),$$ (4)

The general expression for the local field *v*_*i*_ then reads:

$$v_{i}=\sum_{j=1}^{N}w_{ij}s_{j}+x_{i}-I\left(\vec{x},\vec{s}\right).$$ (5)

In the absence of external fields, *x*_*i*_ = 0 for all *i*, and thus Eqs 4 and 5 reduce to the previous expressions Eqs 3 and 2.

The goal of the learning process is to find values of *w*_*ij*_’s such that the patterns $\left\{\vec{\xi^{\mu }}\right\}$ become attractors of the network dynamics. Qualitatively, this means that, if the training process is successful, then whenever the network state gets sufficiently close to one of the stored patterns, i.e. whenever the Hamming distance $d=\sum_{i=1}^{N}|\xi_{i}^{\mu }-s_{i}|$ between the current network state and a pattern *μ* is sufficiently small, the network dynamics in the absence of external inputs should drive the network state towards a fixed point equal to the pattern itself (or very close to it). The general underlying idea is that, after a pattern is successfully learned, some brief external input which initializes the network close to the learned state would be sufficient for the network to recognize and retrieve the pattern. The maximum value of *d* for which this property holds is then called the basin of attraction size (or just basin size hereafter for simplicity); indeed, there is generally a trade-off between the number of patterns which can be stored according to this criterion and the size of their basin of attraction.

More precisely, the requirement that a pattern $\vec{\xi^{\mu }}$ is a fixed point of the network dynamics in the absence of external fields can be reduced to a condition for each neuron *i* (cfr. Eqs 4 and 5):

$$\forall i:\;\Theta (\sum_{j=1}^{N}w_{ij}\xi_{j}^{\mu }-I(\vec{0},\vec{\xi^{\mu }})-\theta )=\xi_{i}^{\mu }.$$ (6)

This condition only guarantees that, if the network is initialized into a state $\vec{s}\;=\;\vec{\xi^{\mu }}$, then it will not spontaneously change its state, i.e. it implements a zero-size basin of attraction. A simple way to enlarge the basin size is to make the requirement in Eq 6 more stringent, by enforcing a more stringent constraint for local fields:

$$\begin{array}{c} \forall i:\;\{\begin{array}{cc} \sum_{j=1}^{N}w_{ij}\xi_{j}^{\mu }-I(\vec{0},\vec{\xi^{\mu }})>\theta +f\sqrt{N}\epsilon & \text{if}\;\xi_{i}^{\mu }=1\\ \sum_{j=1}^{N}w_{ij}\xi_{j}^{\mu }-I(\vec{0},\vec{\xi^{\mu }})<\theta -f\sqrt{N}\epsilon & \text{if}\;\xi_{i}^{\mu }=0, \end{array} \end{array}$$ (7)

where *ϵ* ≥ 0 is a robustness parameter. When *ϵ* = 0, we recover the previous zero-basin-size scenario; increasing *ϵ* we make the neurons’ response more robust towards noise in their inputs, and thus we enlarge the basin of attraction of the stored patterns (but then fewer patterns can be stored, as noted above).

### The three-threshold learning rule (3TLR)

In the training phase, the network is presented with patterns as strong external fields *x*_*i*_. Patterns are presented sequentially in random order. For each pattern *μ*, we simulated the following scheme:

**Step 1**: The pattern is presented (i.e. the external inputs *x*_*i*_ are set to $X\xi_{i}^{\mu }$). A single step of synchronous updating is performed (Eqs 1, 4 and 5). If the external inputs are strong enough, i.e. *γ* is large enough, this updating sets the network in a state corresponding to the presented pattern.
**Step 2**: Learning occurs. Each neuron *i* may update its synaptic weights depending on 1) their current value $w_{ij}^{t}$, 2) the state of the pre-synaptic neurons, and 3) the value of the local field *v*_*i*_. Therefore, all the information required is locally accessible, and no explicit error signals are used. The new synaptic weights $w_{ij}^{t+1}$ are set to:
  $$w_{ij}^{t+1}\;=\;\{\begin{array}{ll} w_{ij}^{t}-\eta s_{j}, & \text{if}\;\theta_{0}<v_{i}<\theta \\ w_{ij}^{t}+\eta s_{j}, & \text{if}\;\theta <v_{i}<\theta_{1}\\ w_{ij}^{t}, & \text{otherwise,} \end{array}$$ (8)
  where *η* is the learning rate, and *θ*_0_ and *θ*_1_ are two auxiliary learning thresholds set as
  $$\begin{array}{ccc} \theta_{0} & = & \theta -(\gamma +\epsilon )f\sqrt{N} \end{array}$$ (9)
  $$\begin{array}{ccc} \theta_{1} & = & \theta +(\gamma +\epsilon )f\sqrt{N}. \end{array}$$ (10)

We refer to this update scheme as the “three-threshold learning rule” (3TRL). After some number of presentations, we checked whether the patterns are learned by presenting a noisy version of these patterns, and checking whether the patterns (or network states which are very close to the patterns) are fixed points of the network dynamics.

**Fig 2:**
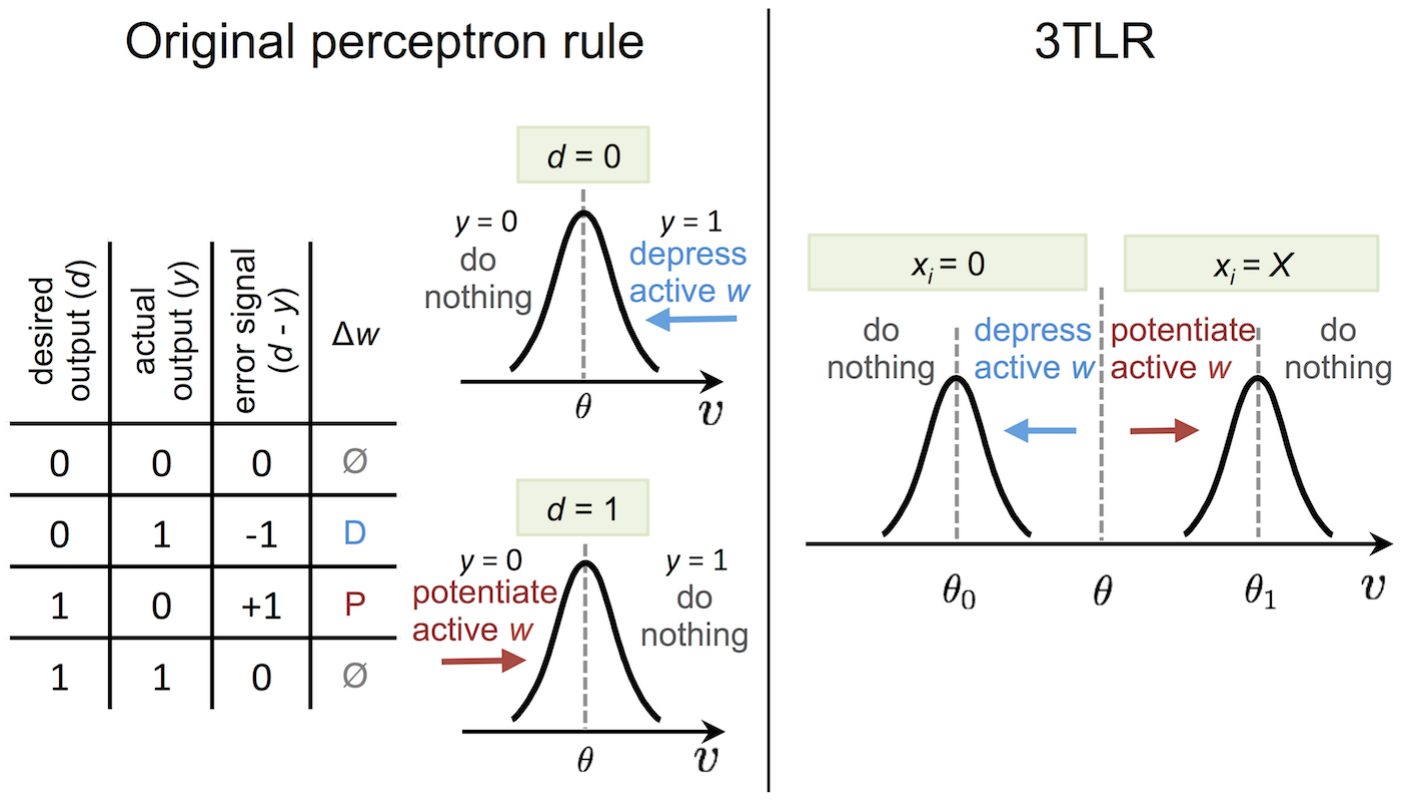
The three-threshold learning rule (3TLR), and its relationship with the standard perceptron learning rule (PLR). The perceptron learning rule modifies the synaptic weights by comparing the desired output with the actual output to obtain an error signal, subsequently changing the weights in the opposite direction of the error signal (see the table in the left panel). For a pattern which is uncorrelated with the current synaptic weights, the distribution is Gaussian (in the limit of large *N*), due to the central limit theorem. *H*_0_ is set such that, on average, a fraction *f* of the local fields are above the neuronal threshold *θ*; in the case of *f* = 0.5, this means that the Gaussian is centered on *θ* (left panel). In our model (Fig 1B), the desired output is given as a strong external input, whose distribution across the population is bimodal (with two delta functions on *x*_*i*_ = 0 and *x*_*i*_ = *X*); therefore, the distribution of the local fields during stimulus presentation becomes bimodal as well (right panel). The left and right bumps of this distribution correspond to cases where the desired outputs are zero and one, respectively. Note that, since the external input also elicits an inhibitory response, the neurons in the network which are not directly affected by the external input (i.e. those with desired output equal to zero) are effectively hyperpolarized. If *X* is sufficiently large, the two distributions do not overlap, and the four cases of the PLR can be mapped to the four regions determined from the three thresholds, indicated by vertical dashed lines (see text).

When *N* ≫ 1, *γ* is large enough, and *H*_1_ = *fX*, the update rule described by Eq 8 is essentially equivalent to the perceptron learning rule for the task described in Eq 7. This can be shown as follows (see also Fig 2 for a graphical representation of the case *f* = 0.5 and *ϵ* = 0): when a stimulus is presented, the population of neurons is divided in two groups, one for which *x*_*i*_ = 0 and one for which *x*_*i*_ = *X*. The net effect of the stimulus presentation on the local field has to take into account the indirect effect through the inhibitory part of the network (see Eq 4), and thus is equal to −*fX* for the *x*_*i*_ = 0 population and to (1 − *f*)*X* for the *x*_*i*_ = *X* population. Before learning, the distribution of the local fields across the excitatory population, in the limit *N* → ∞, is a Gaussian whose standard deviation is proportional to $\sqrt{N}$, due to the central limit theorem; moreover, the parameter *H*_0_ is set so that the average activity level of the network is *f*, which means that the center of the Gaussian will be within a distance of order $\sqrt{N}$ from the neuronal threshold *θ* (this also applies if we use different values for the spontaneous activity level and the pattern activity level). Therefore, if $X=\gamma \sqrt{N}$ is large enough, the state of the network during stimulus presentation will be effectively clamped to the desired output, i.e. $s_{i}=\xi_{i}^{\mu }$ for all *i*. This fact has two consequences: 1) the local field potential can be used to detect the desired output by just comparing it to the threshold, and 2) each neuron *i* will receive, as its recurrent inputs {*s*_*j*_}_*j* ≠ *i*_, the rest of the pattern ${\left\{\xi_{j}^{\mu }\right\}}_{j\neq i}$. Furthermore, due to the choice of the secondary thresholds *θ*_0_ and *θ*_1_ in Eqs 9 and 10, the difference between the local field and *θ*_0_ (or *θ*_1_) during stimulus presentation for the *x*_*i*_ = 0 population (or *x*_*i*_ = *X*, respectively) is equal to the difference between the local field and $\theta -f\sqrt{N}\epsilon$ (or $\theta +f\sqrt{N}\epsilon$, respectively) in the absence of external stimuli, provided the recurrent inputs are the same. Therefore, the value of the local field *v*_*i*_ during stimulus presentation in relation to the three thresholds *θ*, *θ*_0_ and *θ*_1_ is sufficient to determine whether an error is made with respect to the constraints of Eq 7, and which kind of error is made. Following these observations, it is straightforward to map the standard perceptron learning rule on the 4 different cases which may occur (see Fig 2), resulting in Eq 8.

**Fig 3:**
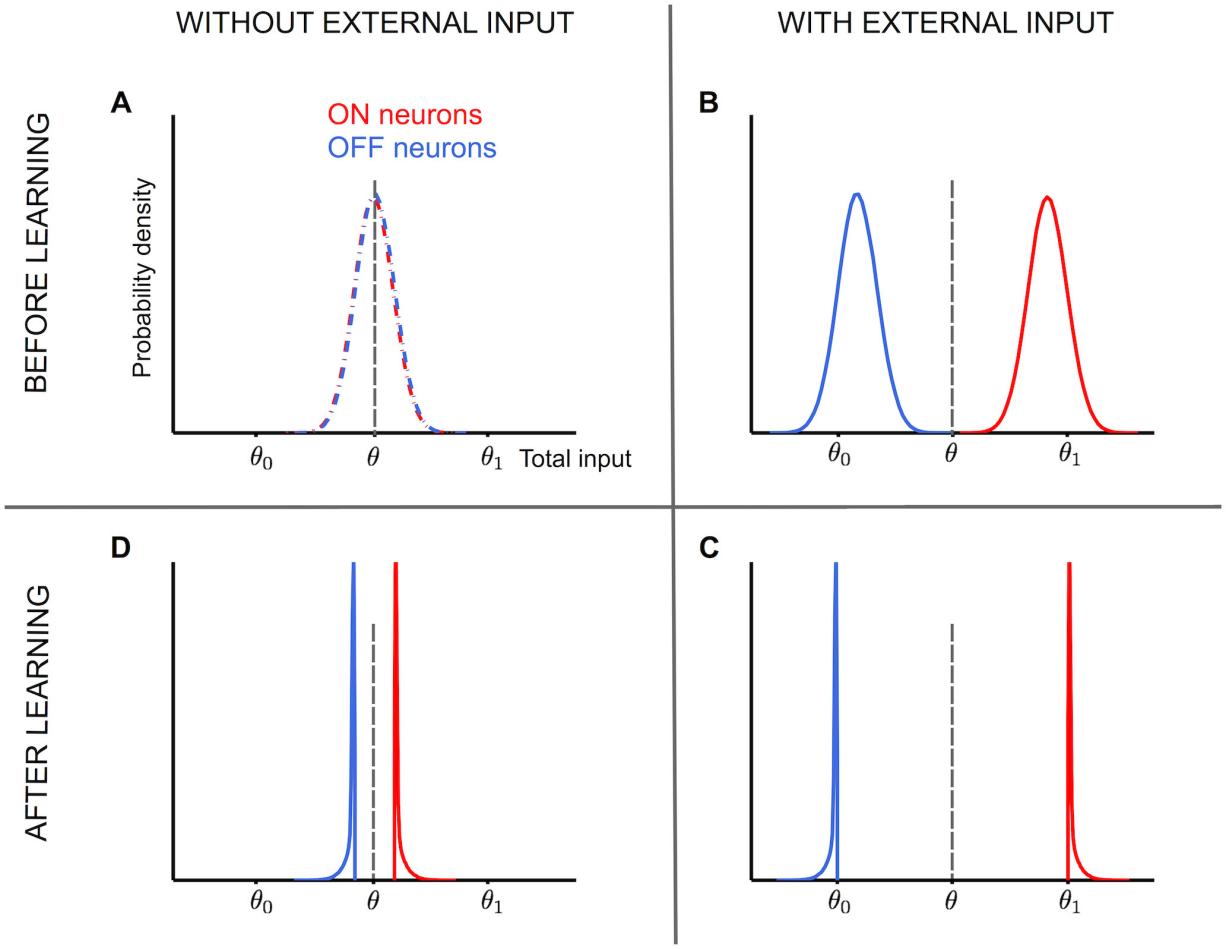
Distribution of local fields before and after learning for *f* = 0.5 and non-zero robustness. **A.** Before learning begins, the distribution of local field of neurons is a Gaussian distribution (due to central limit theorem) centered around neuronal threshold *θ* both for neurons with the desired output zero (OFF neurons) and with the desired output one (ON neurons). The goal is to have the local field distribution of ON neurons (red curve) to be above the threshold *θ*, and that of OFF neurons to be below *θ*. **B.** Once any of the to-be-stored patterns are presented as strong external fields, right before the learning process starts, the local field distribution of the OFF neuron shifts toward the left-side centered around $\theta_{0}+f\epsilon \sqrt{N}$, whereas the distribution of the ON neurons moves toward the right-side, centered around $\theta_{1}-f\epsilon \sqrt{N}$, with a negligible overlap between the two curves if the external field is strong enough. Thanks to the strong external fields and global inhibition, the local fields of the ON and OFF neurons are well separated. **C.** Due to the learning process, the local fields within the depression region [i.e. (*θ*_0_, *θ*)] get pushed to the left-side, below *θ*_0_, whereas those within the potentiation region get pushed further to the right-side, above *θ*_1_. If the learning process is successful, it will result in a region (*θ*_0_, *θ*_1_) which no longer contain local fields, with two sharp peaks on *θ*_0_ and *θ*_1_. **D.** After successful learning, once the external fields are removed, the blue and red curves come closer, with a gap equal to $2f\epsilon \sqrt{N}$. The larger the robustness parameter *ϵ*, the more the gap between the left- and right-side of the distribution. Notice that now the red curve is fully above *θ* which means those neurons remain stably ON, while the the blue curve is fully below *θ*, which means those neurons are stably OFF. Therefore the corresponding pattern is successfully stored by the network.

In Fig 3 we demonstrate the effect of the learning rule on the distribution of the local field potentials as measured from a simulation (with *f* = 0.5 and *ϵ* = 1.2): the initial distribution of the local fields of the neurons, before the learning process takes place and in the absence of external fields, is well described by a Gaussian distribution centered on the neuronal threshold *θ* (see Fig 3A) with a standard deviation which scales as $\sqrt{N}$. During a pattern presentation, the resulting distribution becomes a bimodal one; before learning takes place, the distribution is given by the sum of two Gaussians of equal width, centered around $\theta_{0}+f\sqrt{N}\epsilon$ and $\theta_{1}-f\sqrt{N}\epsilon$ (Fig 3B). The left Gaussian corresponds to the cases where *x*_*i*_ = 0 and the right one to the cases where *x*_*i*_ = *X*. Having applied the learning rule, we observe that the depression region (i.e. the interval (*θ*_0_, *θ*)) and the potentiation region (i.e. (*θ*, *θ*_1_)) gets depleted (Fig 3C). In the testing phase, when the external inputs are absent, the left and right parts of the distribution come closer, such that the distance between the two peaks is equal to at least $2\epsilon f\sqrt{N}$ (Fig 3D). This margin between the local fields of the ON and OFF neurons makes the attractors more robust.

### Storage capacity

Since our proposed learning rule is able to mimic (or approximate, depending on the parameters) the perceptron learning rule, which is known to be able to solve the task posed by Eq 7 whenever a solution exists, we expect that a network implementing such rule can get close to maximal capacity in terms of the number of memories which it can store at a given robustness level. The storage capacity, denoted by *α* = *p*/*N*, is measured as a ratio of the maximum number of patterns *p* which can successfully be stored to the number of neurons *N*, in the limit of large *N*. As mentioned above, it is a function of the basin size.

We used the following definition for the basin size: a set of *p* patterns is said to be successfully stored at a size *b* if, for each pattern, the retrieval rate when starting from a state in which a fraction *b* of the pattern was randomized is at least 90%. The retrieval rate is measured by the probability that the network dynamics is able to bring the network state to an attractor within 1% distance from the pattern, in at most 30 steps. The distance between the state of the network and a pattern *μ* is measured by the normalized Hamming distance $\frac{1}{N}\sum_{i=1}^{N}|s_{i}-\xi_{i}^{\mu }|$. Therefore, at coding level *f* = 0.5, reaching a basin size *b* means that the network can successfully recover patterns starting from a state at distance *b*/2.

**Fig 4:**
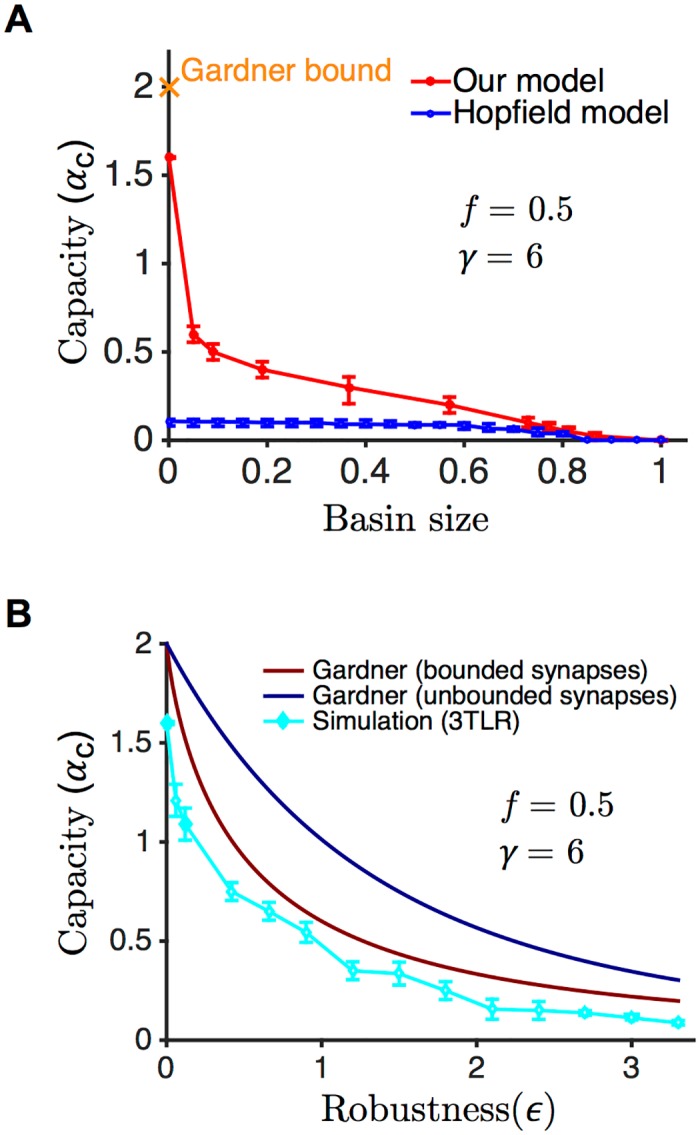
Critical capacity as a function of the basin size and the robustness parameter. **A.** The red plot shows the critical capacity as a function of the size of the basins of attraction (*N* = 1001 neurons in the dense regime *f* = 0.5) when the strength of the external field is large (*γ* = 6) such that the ON and OFF neuronal populations are well separated. The points indicate 0.5 probability of successful storage at a given basin size, optimized over the robustness parameter *ϵ*. The error bars show the [0.95,0.05] probability interval for successful storage. The blue plot shows the performance of the Hopfield model with *N* = 1001 neurons. The maximal capacity at zero basin size (the Gardner bound) is equal to 2. **B.** To compare the result of simulation of our model with the analytical results, we plotted the critical capacity as a function of the robustness parameter *ϵ*. The dark red curve is the critical capacity versus *ϵ* for our model obtained form analytical calculations (see Materials and Methods), the cyan line shows the result of simulations of our model, and the dark blue shows the Gardner bound for a network with no constraints on synaptic weights. The difference between the two theoretical curves is due to the constraints on the weights in our network.

Fig 4A shows the maximal capacity as a function of the basin size for a simulated network of *N* = 1001 neurons. We simulated many pairs of (*α*, *ϵ*) with different random seeds, obtaining a probability of success for each pair. The red line shows the points for which the probability of successful storage is 0.5, and the error bars span 0.95 to 0.05 success probability. The capacity was optimized over the robustness parameter *ϵ*. The maximal capacity (the Gardner bound) in the limit of *N* → ∞ at the zero basin size is *α*_*c*_ = 2 for our model (see Materials and Methods for the calculation), as for a network with unconstrained synaptic weights [20]. In Fig 4A, we also compare our network with the Hopfield model. Our network stores close to the maximal capacity at zero basin size, at least eleven times more than the Hopfield model. Across the range of basin sizes, 3TLR achieves more than twice the capacity that can be achieved with the Hopfield model.

The enlargement of the basin of attraction was achieved by increasing the robustness parameter *ϵ*. We computed the maximal theoretical capacity as a function of *ϵ* at *N* → ∞ (see Materials and Methods) and compared it to our simulations, and to the maximal theoretical capacity of the Hopfield network. The results are shown in Fig 4B. For any given value of *ϵ*, the cyan curve shows the maximum *α* for which the success ratio with our network was at least 0.5 across different runs. The difference between the theory and the experiments in our model can be ascribed to several factors: the finite size of the network; the choice of the finite learning rate *η*, and the fact that we imposed a hard limit on the number of pattern presentations (see number of iterations in Table 1), while the perceptron rule for excitatory synaptic connectivity is only guaranteed to be optimal in the limit of *η* → 0, with a number of presentations inversely proportional to *η* [25]. Note that the correspondence between the PLR and the 3TLR is only perfect in the large *γ* limit, and is only approximate otherwise, as can be shown by comparing explicitly the synaptic matrices obtained by both algorithms on the same set of patterns (see Materials and Methods).

**Table 1:** Table of parameters in the simulation.

**Parameter name**	**Value in dense regime**	**Value in sparse regime**
*N*	1001	1001
$\lambda ={\bar{w}}_{ij}^{\text{init}}$	≈ 1.08	≈ 1.08
*f*	0.5	0.2
*ψ*	0.35	0.35
*θ*	350	350
*η*	0.01 [0.001 when *ϵ* = 0]	0.01 [0.001 when *ϵ* = 0]
*γ*	6.0	12.0
# of interations (learning)	1000 [10000 when *ϵ* = 0]	1000 [10000 when *ϵ* = 0]
# of trials in test phase	50	50

**Fig 5:**
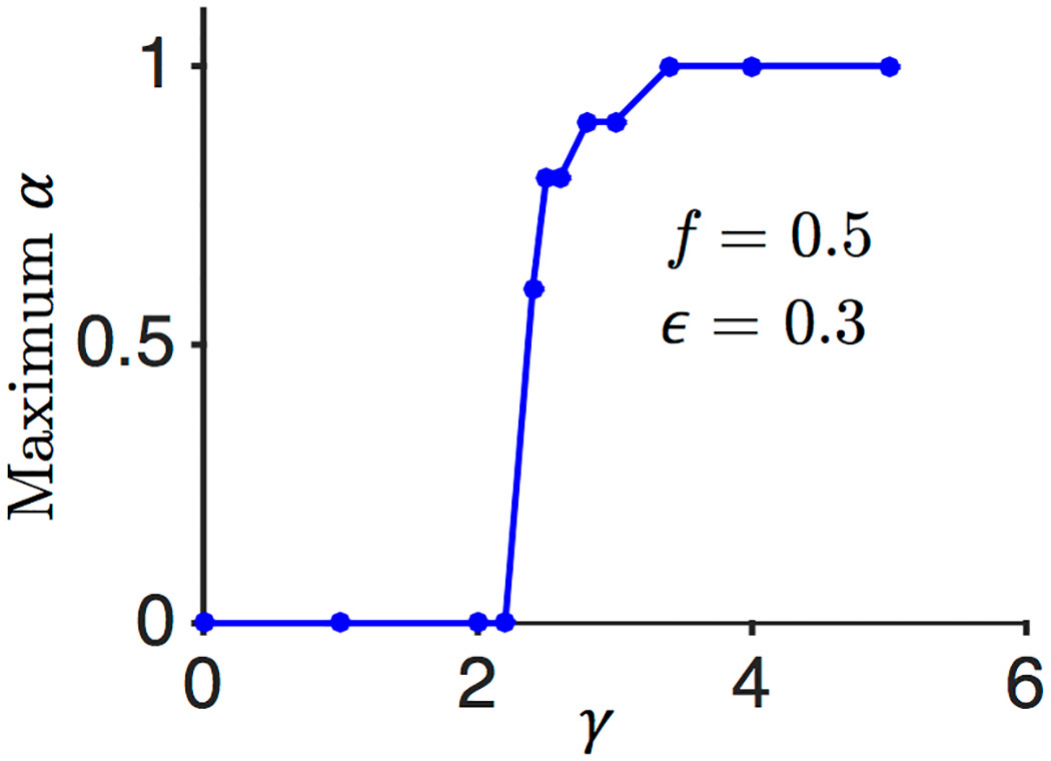
Dependence of the critical capacity on the strength of the external input. We varied the strength of the external field (*γ*) in order to quantify its effect on the learning process. The critical capacity is plotted as a function of *γ* at a fixed robustness *ϵ* = 0.3 in the dense regime *f* = 0.5. The simulations show that there is a very sharp drop in the maximum *α* when *γ* goes below ≈ 2.4.

A crucial ingredient of the 3TLR is having a strong external input which effectively acts as a supervisory signal. How strong do the external fields need to be? How much does the capacity depend on this strength? To answer these questions, we measured the maximum number of stored patterns as a function of the parameter *γ* which determines the strength of external fields as $X=\gamma \sqrt{N}$. This parameter, in fact, determines how far the two Gaussian distributions of the local field are; as shown in Fig 2, the distance between the two peaks of the distribution is *X*. For large enough *γ*, the overlap of these two distributions is negligible and the capacity is maximal; but as we lower *γ*, the overlap increases, causing the learning rule to make mistakes, i.e. when it should potentiate, it depresses the synapses and vice versa. In our simulations with *N* = 1001 neurons in the dense regime *f* = 0.5 at a fixed epsilon *ϵ* = 0.3, we varied *γ* and computed the maximum *α* that can be achieved with a fixed number of iterations (1000). The capacity indeed gradually decreases as *γ* decreases, until it reaches a threshold, below which there is a sharp drop of capacity (see Fig 5). With the above values for the parameters, this transition occurs at *γ* ≈ 2.4.

**Fig 6:**
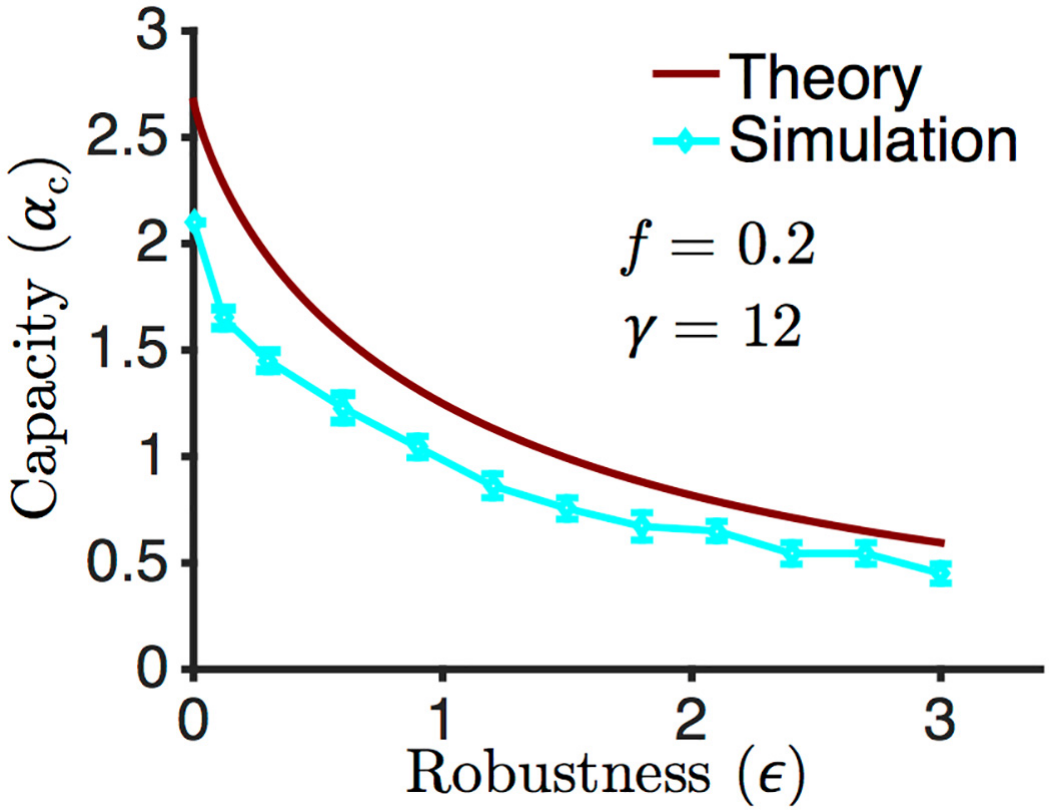
Capacity as a function of the robustness parameter *ϵ* at sparseness *f* = 0.2. The theoretical calculations is compared with the simulations for *f* = 0.2. Note that the capacity in the sparse regime is higher than in the dense regime.

The 3TLR can also be adapted to work in a sparser regime, at a coding level lower than 0.5. However, the average activity level of the network is determined by *H*_0_, and their relationship also involves the variance of the distribution of the synaptic weights when *f* ≠ 0.5 (see Materials and Methods). During the learning process, the variance of the weights changes, which implies that the parameter *H*_0_ must adapt correspondingly. In our simulations, this adaptation was performed after each complete presentation of the whole pattern set. In practice, this additional self-stabilizing mechanism could still be performed in an unsupervised fashion along with (or in alternation with) the learning process. Using this adjustment, we simulated the network at *f* = 0.2 and compared the results with the theoretical calculations. As shown in Fig 6, we can achieve at least 70% of the critical capacity across different values of the robustness parameter *ϵ*.

**Fig 7:**
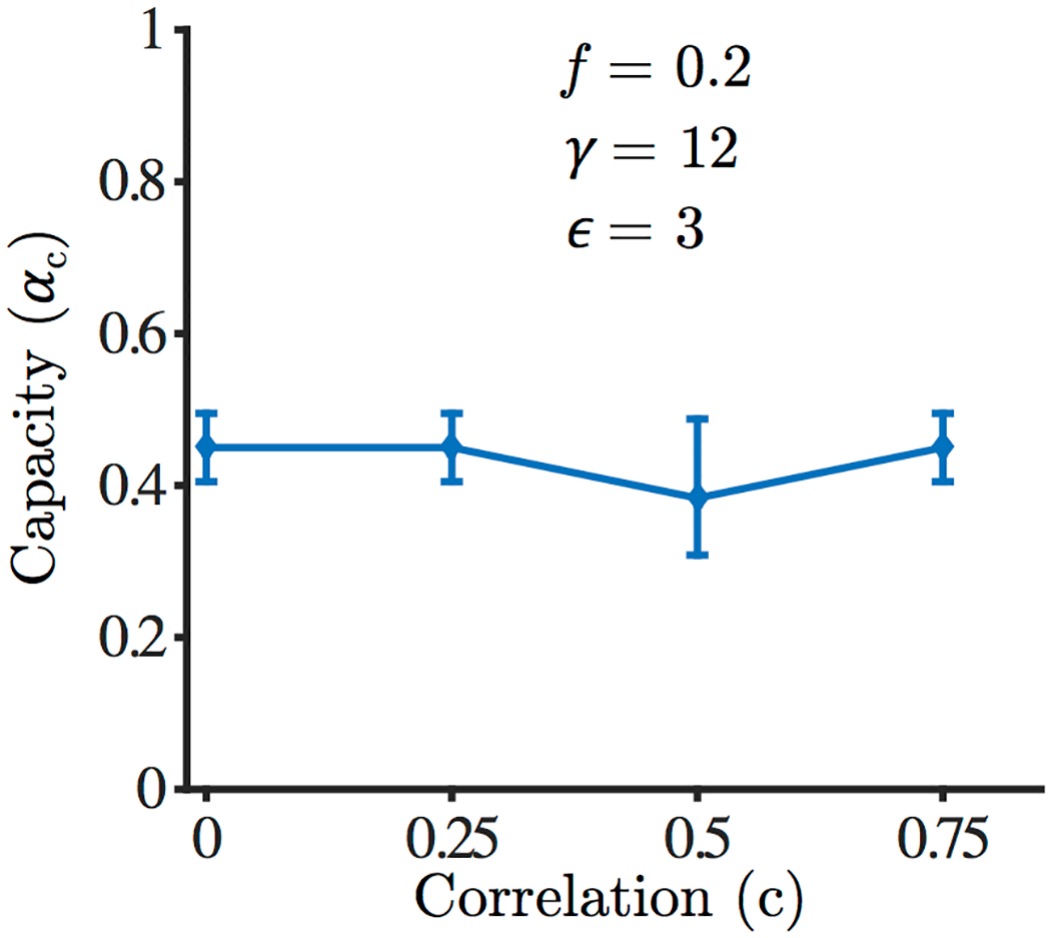
Capacity as a function of correlations in the input patterns, for *f* = 0.2 at *ϵ* = 3.0. Patterns are organized in categories, with a correlation *c* with the prototype of the corresponding category (see text).

We also investigated numerically the effect of correlations in the input patterns. The PLR is able to learn correlated patterns as long as a solution to the learning problem exists. As the 3TLR approximates the PLR, we expect the 3TLR to be able to learn correlated patterns as well. As a simple model of correlation, we tested patterns organized in *L* categories [26, 27]. Each category was defined by a randomly generated prototype. Prototypes were uncorrelated from category to category. For each category, we then generated *p*/*L* patterns independently with a specified correlation coefficient *c* with the corresponding prototype. We show in Fig 7 the results of simulations with *L* = 5, *f* = 0.2 and *ϵ* = 3. The figure shows that the learning rule reaches a capacity that is essentially independent of *c*, in the range 0 ≤ *c* ≤ 0.75.

### Statistical properties of the connectivity matrix

**Fig 8:**
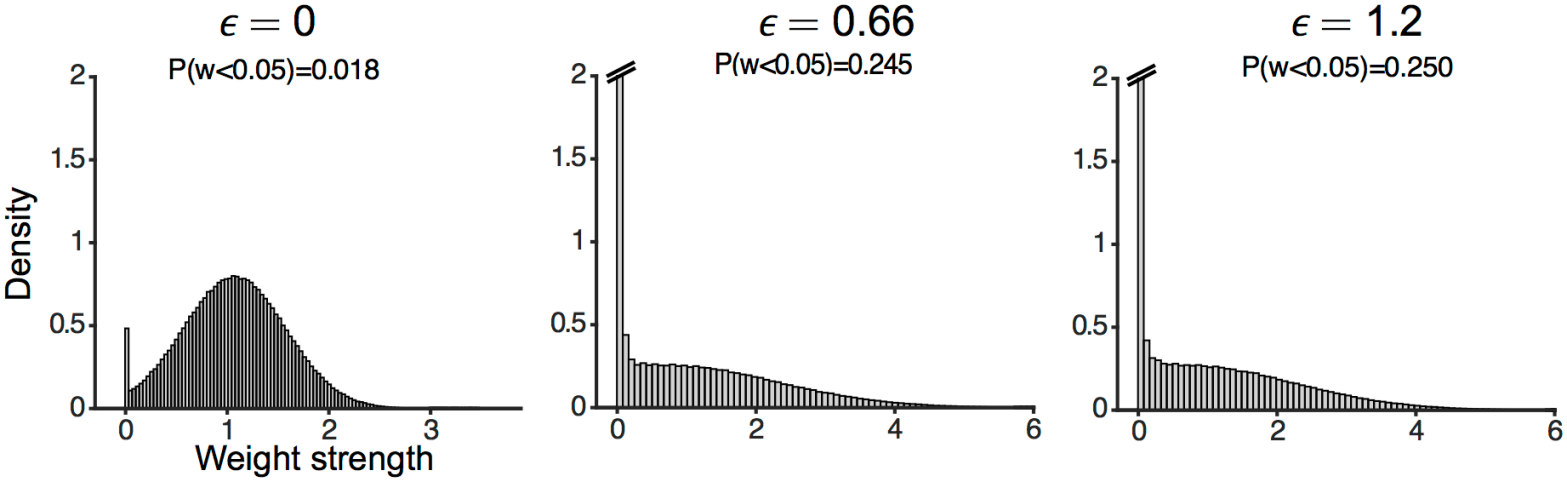
Synaptic weight distributions. Comparing the distributions of the synaptic weights at critical capacity for three different values of robustness obtained from simulation. The distribution of weights approaches a Dirac-delta distribution at zero plus a truncated Gaussian. As the patterns become more robust, the center of the partial Gaussian shifts towards the left, and the number of silent synapses increases.

We next investigated the statistical properties of the connectivity matrix after the learning process. Previous studies have shown that the distribution of synaptic weights in perceptrons with excitatory synapses becomes at maximal capacity a delta function at zero weight, plus a truncated Gaussian for strictly positive weights [25, 28–30]. Our model differs from this setting because of the global inhibitory feedback. Despite this difference, the distribution of weights in our network bear similarities with the results obtained in these previous studies: the distribution exhibits a peak at zero weight (‘silent’, or ‘potential’ synapses), while the distribution of strictly positive weights resembles a truncated Gaussian. Finally, the fraction of silent synapses increases with the robustness parameter (see Fig 8).

**Fig 9:**
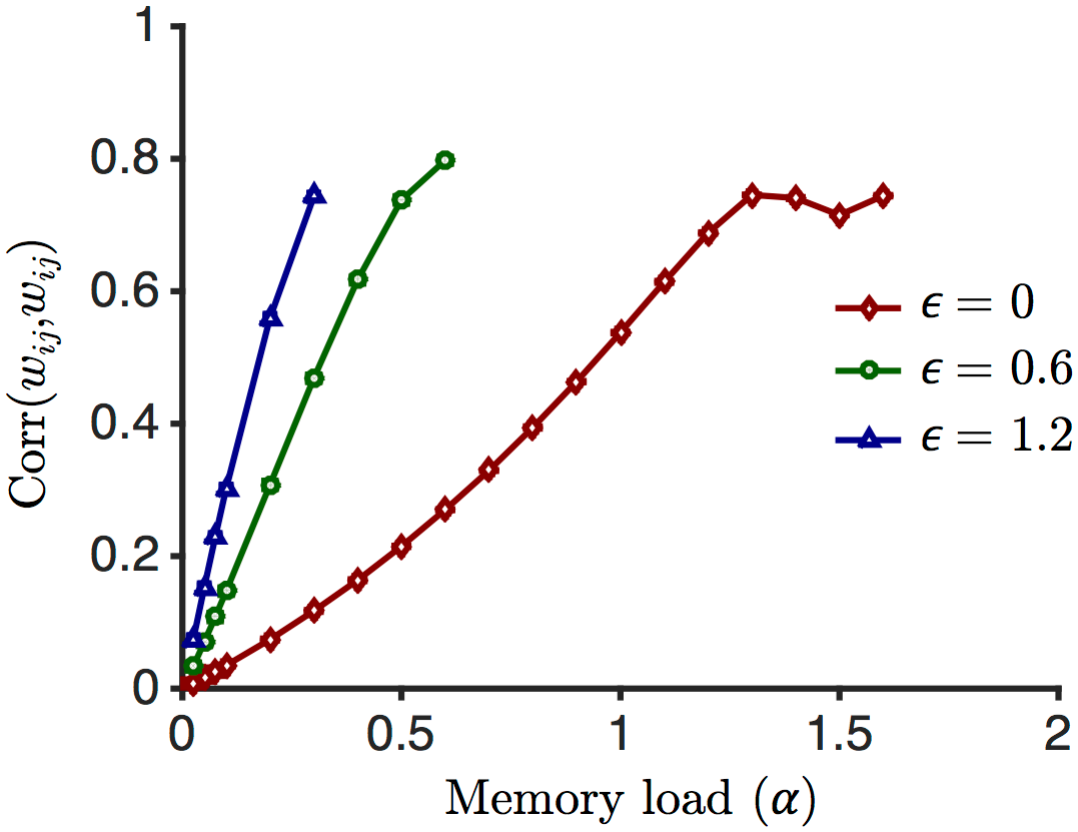
The degree of symmetry of the weight matrix. The Pearson correlation coefficient between *w*_*ij*_ and *w*_*ji*_ is computed at different values of *α* for three values of *ϵ*. As *α* increases the weight matrix tends to be more symmetric, but gets saturated for high *α*. For the same values of *α*, as the robustness increases, the correlation also increases, so the weight matrix becomes more symmetric. Error bars (across 10 runs) are smaller than the symbols.

We have also computed the degree of symmetry of the weight matrix. The symmetry degree is computed as the Pearson correlation coefficient between the reciprocal weights in pairs of neurons. We observe a general trend towards an increasingly symmetric weight matrix as more patterns are stored, for all values of the robustness parameter *ϵ* (see Fig 9).

## Discussion

We presented a biologically-plausible learning rule that is characterized by three thresholds, and is able to store memory patterns close to the maximal storage capacity in a recurrent neural networks without the need of an explicit “error signal”. We demonstrated how the learning rule can be considered a transformed version of the PLR in the limit of a strong external field. Our network implements the separation between excitatory and inhibitory neurons, with learning occurring only at excitatory-to-excitatory synapses. We simulated a recurrent network with *N* = 1001 binary neurons, reaching to *α*_*c*_ = 1.6 at zero basin size. We then used a robustness parameter *ϵ* to enlarge the basin size. The simulations showed that we are close to the theoretical capacity across the whole investigated range of values of *ϵ*. We expect that as *N* increases and the learning rate gets smaller, this difference would go to zero.

Two crucial ingredients of the 3TLR are necessary: (1) strong external inputs, (2) three learning thresholds which are set according to the statistics of inputs to the neuron. The learning rule only uses information that is local to a synapse and corresponding neurons. Like classic Hebbian learning rules, our 3TLR works in an online fashion. In addition, it can also perform as a ‘palimpsest’ [31–33]: in case the total number of patterns exceeds the maximal capacity (at a certain basin size) the network begins to forget patterns that are not being presented anymore.

### Comparison with other learning rules

The 3TLR can be framed in the setting of the classic Bienenstock-Cooper-Munro (BCM) theory [34, 35], with additional requirements to adapt it to the attractor network scenario. The original BCM theory uses firing-rate units, and prescribes that synaptic modifications should be proportional to (1) the synaptic input, and (2) a function *ϕ*(*v*) of the total input *v* (or, equivalently, of the total output). The function *ϕ*(*v*) is subject to two conditions: (1) *ϕ*(*v*) ≥ 0 (or ≤ 0) when *v* > *θ* (or < *θ*, respectively); (2) *ϕ*(0) = 0. The parameter *θ* is also assumed to change, but on a longer time scale (such that the changes reflect the statistics of the inputs); this (metaplastic) adaptation has the goal of avoiding the trivial situations in which all inputs elicit indistinguishable responses. This (loosely specified) framework ensures that, under reasonable conditions, the resulting units become highly selective to a subset of the inputs, and has been mainly used to model the developmental stages of primary sensory cortex. The arising selectivity is spontaneous and completely unsupervised: in absence of further specifications, the units become selective to a random subset of the inputs (e.g. depending on random initial conditions).

Our model is defined on simpler (binary) units; however, if we define *ϕ*(*v*) = Θ (*v* − *θ*) Θ (*θ*_1_ − *v*) − Θ (*θ* − *v*) Θ (*v* − *θ*_0_), then *ϕ* behaves according to the prescriptions of the BCM theory. Furthermore, we have essentially assumed the same slow metaplastic adaptation mechanism of BCM, even though we have assigned this role explicitly to the inhibitory part of the network (see Materials and Methods). On the other hand, our model has additional requirements: (1) *ϕ*(*v*) = 0 when *v* < *θ*_0_ or *v* > *θ*_1_, (2) plasticity occurs during presentation of external inputs, which in turn are strong enough to drive the network towards a desired state. The second requirement ensures that the network units become selective to a specific subset of the inputs, as opposed to a random subset as in the original BCM theory, and thus that they are able to collectively behave as an attractor network. The first requirement ensures that each unit operates close to critical capacity. Indeed, these additional requirements involve extra parameters with respect to the BCM theory, and we implicitly assume these parameters to also slowly adapt according to the statistics of the inputs during network formation and development.

A variant of the BCM theory, known as ABS rule [36, 37] introduced a lower threshold for LTD, analogous to our *θ*_0_, motivated by experimental evidence; however, a high threshold for LTP, analogous to our *θ*_1_, was not used there, or—to our knowledge—in any other BCM variant. The idea of stopping plasticity above some value of the ‘local field’ has been introduced previously to stabilize the learning process in feed-forward networks with discrete synapses [38–40]. Our study goes beyond these previous works in generalizing such a high threshold to recurrent networks, and showing that the resulting networks achieve close to maximal capacity.

### Comparison with data and experimental predictions

In vitro experiments have characterized how synaptic plasticity depends on voltage [41] and firing rate [42], both variables that are expected to have a monotonic relationship with the total excitatory synaptic inputs received by a neuron. In both cases, a low value of the controlling variable leads to no changes; intermediate values lead to depression; and high values to potentiation. These three regimes are consistent with the three regions for *v* < *θ*_1_ in Fig 2. The 3TLR predicts that a fourth region should occur at sufficiently high values of the voltage and/or firing rates. Most of the studies investigating the dependence of plasticity on firing rate or voltage have not reported a decrease in plasticity at high values of the controlling variables, but these studies might have not increased sufficiently such variables. To our knowledge, a single study has found that at high rates, the plasticity vs rate curve is a decreasing function of the input rate [43].

Another test of the model consists in comparing the statistics of the synaptic connectivity with experimental data. As it has been argued in several recent studies [25, 28, 30, 44, 45], networks with plastic excitatory synapses are generically sparse close to maximal capacity, with a connection probability that decreases with the robustness of information storage, consistent with short range cortical connectivity [46, 47]. Our network is no exception, though the fraction of silent synapses that we observe is significantly lower than in models that lack inhibition. Furthermore, network that are close to maximal capacity tends to have a connectivity matrix that has a significant degree of symmetry, as illustrated by the over-representation of bidirectionally connected pairs of neurons, and the tendency of bidirectionally connected pairs to form stronger synapses than unidirectionally connected pairs as observed in cortex [47, 48], except in barrel cortex [49]. Again, the 3TLR we have proposed here reproduces this feature (Fig 9), consistent with the fact that the rule approaches the optimal capacity.

### Future directions

Our network uses the simplest possible single neuron model [50]. One obvious direction for future work would be to implement the learning rule in a network of more realistic neuron models such as firing rate models or spiking neuron models. Another potential direction would be to understand the biophysical mechanisms leading to the high threshold in the 3TLR. In any case, we believe the results discussed here provide a significant step in the quest for understanding how learning rules in cortical networks can optimize information storage capacity.

## Materials and Methods

### Simulation

The main equations of the network, the neuron model, the learning rule, and the criteria for stopping the learning algorithm are outlined in the Results section, Eqs 1–7. We present here additional details about network simulations.

#### Network setup before learning process

Before applying the learning rule, we required the network to have stable dynamics around a desired activity level *f*. A network with only excitatory neurons is highly unstable and typically converges towards the trivial all-off and all-on states; therefore, we implemented a global inhibition such that the network operates around activity level *f*. The basal inhibitory term (*H*_0_) and the inhibitory reaction term (*H*_1_) are defined as:

$$\begin{array}{ccc} H_{0} & = & \left(N-1\right)\left(f\bar{w}-\psi \right)+H^{-1}\left(f\right)\sqrt{\left(N-1\right)f}\sigma_{w} \end{array}$$ (11)

$$\begin{array}{ccc} H_{1} & = & f\gamma \sqrt{N-1} \end{array}$$ (12)

where $H\left(x\right)=\frac{1}{2}\text{erfc}\left(\frac{x}{\sqrt{2}}\right)$ and *H* ^−1^ is the inverse of *H*, *ψ* is defined as *θ* = (*N* − 1)*ψ*; $\bar{w}$ and *σ*_*w*_ are the mean and standard deviation of the synaptic weights, respectively. With these definitions the network dynamics is stable in the sense that the activity level converges to *f* very fast, regardless of the initial condition.

In Eq 11, we see that *H*_0_ depends on the activity level *f* and on the standard deviation of the weights *σ*_*w*_. In the dense regime, *f* = 0.5, we have *H* ^−1^(0.5) = 0, therefore the rightmost term of Eq 11 vanishes, which means that in this regime *H*_0_ is independent of *σ*_*w*_. However, in sparser regimes, the network must be endowed with a mechanism to adjust for the changes in standard deviation, otherwise the learning process would bring the network out of the stable state, changing the basal activity level. In contrast, the mean synaptic efficacy $\bar{w}$ does not change significantly during the learning process.

In all our simulations, the initial values for {*w*_*ij*_} were sampled from a Gaussian distribution with mean and standard deviation equal to one, after which negative values were set to zero. This has the effect the ${\bar{w}}_{ij}^{\text{init}}$ is slightly higher than one. We also set *w*_*ii*_ = 0 for all *i*.

Table 1 shows the values of the parameters used in the simulations, in the dense and sparse regimes.

#### Direct comparison between the 3TLR and the PLR

**Fig 10:**
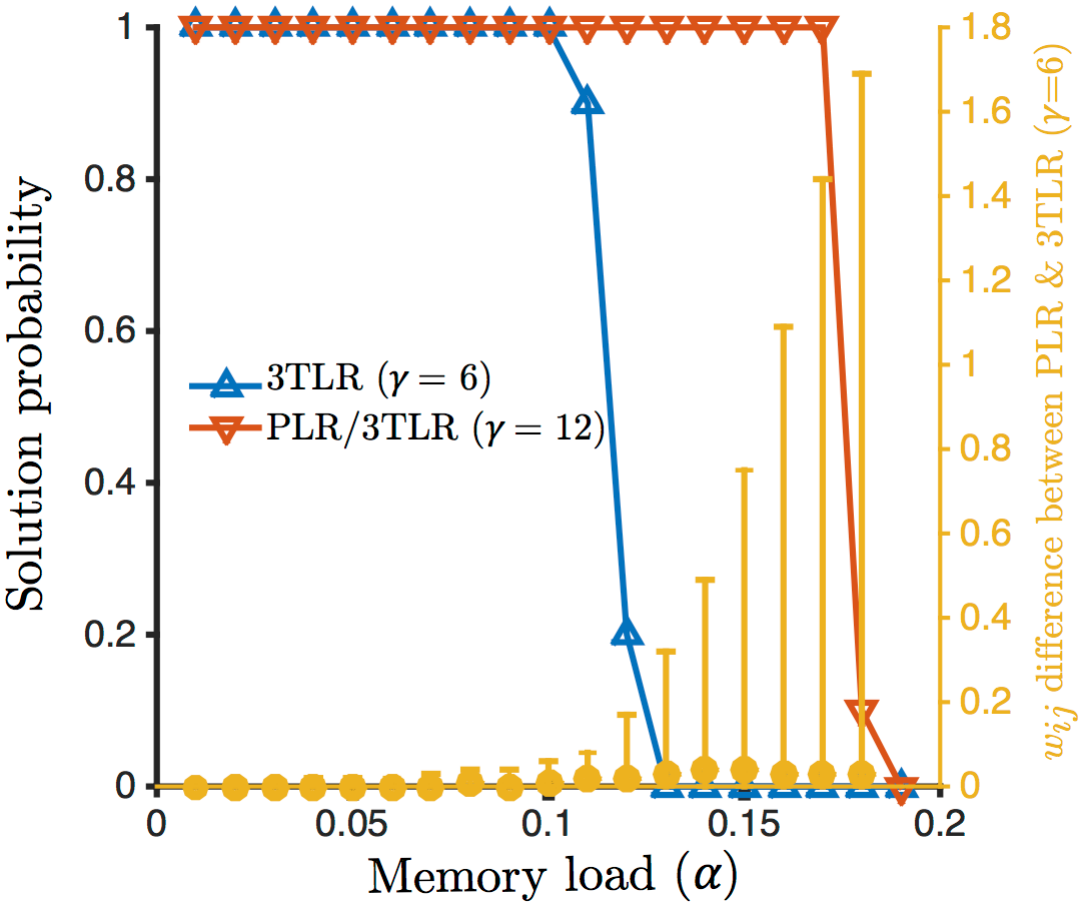
Direct comparions of the 3TLR and the PLR. Success probability for the 3TLR at *γ* = 6 (blue curve, left axis) and the PLR (red curve) at f=0.5 and ϵ=3; the results for the 3TLR at *γ* = 12 are identical to those of the PLR (red curve). The orange points show the absolute difference of weights between the final values of the weights for the PLR at *γ* = 6 and the PLR (right axis): the points show the median of the distribution, while the error bars span the 5th-95th percentiles, showing that, while the distribution is concentrated at near-zero values, outliers appear at the critical capacity of the 3TLR algorithm. (Note that the average value of the weights is in all cases approximately 1.08; also compare the discrepancies with the overall distribution of the weights, Fig 8).

In order to determine the degree to which the 3TLR is able to mimic the PRL, and the effect of deviations from the latter rule, we tested both rules on the same tasks. In these simulations, every part of the simulation code was kept identical—including the pseudo-random numbers used to choose the initial state and the arbitrary permutations for the update order of the units—except for the learning rule. We tested the network in the dense case *f* = 0.5, at *ϵ* = 3, varying the storage load *α*, using 10 samples for each point. We compared the probability of solving the learning task and the distribution of the discrepancies (absolute value of the differences) in the values of the resulting synaptic weights. We tested two values of the parameter *γ*, 6 (as in Fig 4) and 12. We found that at *γ* = 12 there was absolutely no difference between the two rules, while at *γ* = 6 the 3TLR performed slightly worse, and significant deviations from the PLR started to appear close to the maximal capacity of the 3TLR (see Fig 10).

### Analytical calculation of the storage capacity at infinite *N*

#### Entropy calculation

In this section, we present the details of the calculations for the typical storage capacity of our network in the limit of *N* → ∞, using the Gardner analysis [20, 28].

The capacity is defined as the maximum value of *α* = *p*/*N* such that a solution to Eq 7 can typically be found.

We can rewrite Eq 7 as

$$\forall i:\;\prod_{\mu =1}^{\alpha N}\Theta ((2\xi_{i}^{\mu }-1)(\sum_{j=1}^{N}w_{ij}\xi_{i}^{\mu }-H_{0}-\lambda (\sum_{j=1}^{N}\xi_{j}^{\mu }-fN)-\theta )-f\epsilon \sqrt{N})=1$$ (13)

where

$$\begin{array}{ccc} H_{0} & = & Nf\bar{w}-\theta +H^{-1}(f)\sigma_{w}\sqrt{fN} \end{array}$$ (14)

$$\begin{array}{ccc} \lambda & = & \bar{w} \end{array}$$ (15)

Eq 13 becomes:

$$\forall i:\;\prod_{\mu =1}^{\alpha N}\Theta ((2\xi_{i}^{\mu }-1)(\sum_{j=1}^{N}(w_{ij}-\bar{w})\xi_{i}^{\mu }-H^{-1}(f)\sigma_{w}\sqrt{fN})-f\epsilon \sqrt{N})=1$$ (16)

Let us now consider a single unit *i*. We write $\sigma_{i}^{\mu }=\left(2\xi_{i}^{\mu }-1\right)$, and re-parametrize the weights as $W_{ij}=\frac{w_{ij}}{\bar{w}}-1\in \left[-1,\infty \right)$, and also define

$$\begin{array}{ccc} T & = & H^{-1}(f)\sqrt{f} \end{array}$$ (17)

$$\begin{array}{ccc} K & = & \frac{\epsilon }{\bar{w}}. \end{array}$$ (18)

Dropping the index *i* and neglecting terms of order 1, we obtain:

$$\prod_{\mu =1}^{\alpha N}\Theta (\sigma^{\mu }(\sum_{j=1}^{N}W_{j}\xi_{j}^{\mu }-T\frac{\sigma_{w}}{\bar{w}}\sqrt{N})-fK\sqrt{N})=1$$ (19)

Our goal is to compute the quenched entropy of this problem, i.e. the scaled average of the logarithm of the volume of *W* which satisfies the above equation:

$$\begin{array}{ll} S & =\;\frac{1}{N}{\langle \text{log}V\rangle }_{\left\{\xi^{\mu },\sigma^{\mu }\right\}}\\ & =\;\frac{1}{N}{\left\langle \text{log}\int \prod_{j=1}^{N}\left(dW_{j}\Theta \left(W_{j}+1\right)\right)\prod_{\mu =1}^{\alpha N}\Theta \left(\sigma^{\mu }\left(\sum_{j=1}^{N}W_{j}\xi_{j}^{\mu }-\frac{\sigma_{w}}{\bar{w}}T\sqrt{N}\right)-fK\sqrt{N}\right)\right\rangle }_{\left\{\xi^{\mu },\sigma^{\mu }\right\}} \end{array}$$ (20)

The computation proceeds along the lines of [20, 28], by using the so-called replica trick to perform the average of the logarithm of *V*, exploiting the identity:

$$\langle \log V\rangle =\lim_{n\to 0}\frac{\langle V^{n}\rangle -1}{n},$$ (21)

performing the computation for integer values of *n* and using an analytical continuation to perform the limit *n* → 0. We perform the calculation using the replica-symmetric (RS) Ansatz, which is believed to give exact results in the case of perceptron models with continuous weights. The final expression for the entropy depends on six order parameters; the first three are *Q*, *q* and *M*, whose meaning is

$$\begin{array}{ccc} Q & = & \frac{1}{N}\sum_{j}{\left(W_{j}\right)}^{2} \end{array}$$

$$\begin{array}{ccc} q & = & \frac{1}{N}\sum_{j}W_{j}^{a}W_{j}^{b} \end{array}$$

$$\begin{array}{ccc} M & = & \frac{1}{\sqrt{N}}\sum_{j}W_{j} \end{array}$$

where we used *W* ^*a*^ and *W* ^*b*^ to denote two different *replicas* of the system, which can simply be interpreted as two independent solutions to the constraint equation. *Q* is called the self-overlap, and is equal to ${\left(\frac{\sigma_{w}}{\bar{w}}\right)}^{2}$ in our case, while *q* is the mutual-overlap. The remaining order parameters are the conjugate quantities $\hat{Q}$, $\hat{q}$ and $\hat{M}$. The entropy expression is:

$$S(Q,q,M,\hat{Q},\hat{q},\hat{M})=-(Q\hat{Q}-\frac{q\hat{q}}{2})+\alpha Z_{A}(Q,q,M)+Z_{W}(\hat{Q},\hat{q},\hat{M})$$ (22)

where

$$Z_{A}\left(Q,q,M\right)\;=\;\int Du{\left\langle \text{ln}\left(H\left(\frac{K-\sigma \left(M-T\sqrt{Q}\right)+u\left(1-f\right)\sqrt{q}}{\left(1-f\right)\sqrt{Q-q}}\right)\right)\right\rangle }_{\sigma }$$ (23)

$$\begin{array}{ccc} Z_{W}\left(\hat{Q},\hat{q},\hat{M}\right) & = & {\int^{\text{​}}}^{\text{​}}Du\;\ln\left(\int_{-1}^{\infty }\text{d}W\;exp\left(-\frac{1}{2}\left(\hat{q}-2\hat{Q}\right)W^{2}+W\left(u\sqrt{\hat{q}}-\hat{M}\right)\right)\right). \end{array}$$ (24)

We used the usual notation $Du\equiv du\;\frac{e^{-\frac{u^{2}}{2}}}{\sqrt{2\pi }}=du\;G\left(u\right)$ to denote Gaussian integrals, and defined $H\left(x\right)=\int_{x}^{\infty }Du=\frac{1}{2}\text{erfc}\left(\frac{x}{\sqrt{2}}\right)$. In the following, we will also use the shorthand $𝒢\left(x\right)=\frac{G\left(x\right)}{H\left(x\right)}$. We also used the notation ⟨ ⋅ ⟩_*σ*_ to denote the average over the output *σ*, i.e. ⟨*φ*(*σ*)⟩_*σ*_ = *fφ*(1) + (1 − *f*) *φ* (−1) for any function *φ*. The value of the order parameters is found by extremizing *S*. The notation and the following computations can be simplified using:

$$\begin{array}{ccc} \Delta Q & = & Q-q \end{array}$$ (25)

$$\begin{array}{ccc} t_{\sigma }(u) & = & \frac{K-\sigma (M-T\sqrt{Q})+u(1-f)\sqrt{q}}{(1-f)\sqrt{\Delta Q}} \end{array}$$ (26)

$$\begin{array}{ccc} \Delta \hat{Q} & = & \hat{q}-2\hat{Q} \end{array}$$ (27)

$$\begin{array}{ccc} \nu (u,W) & = & e^{-\frac{1}{2}\Delta \hat{Q}W^{2}+W(u\sqrt{\hat{q}}-\hat{M})} \end{array}$$ (28)

The extremization of *S* then results in the system of equations:

$$\Delta \hat{Q}\;=\;\frac{\alpha }{\sqrt{\left(Q-\Delta Q\right)\Delta Q}}\int Du\;u{\langle G\left(t_{\sigma }\left(u\right)\right)\rangle }_{\sigma }$$ (29)

$$\hat{q}\;=\;\frac{\alpha }{\Delta Q}\int Du{\langle G\left(t_{\sigma }\left(u\right)\right)t_{\sigma }\left(u\right)\rangle }_{\sigma }+\Delta \hat{Q}$$ (30)

$$0\;=\;\int Du{\langle G\left(t_{\sigma }\left(u\right)\right)\sigma \rangle }_{\sigma }$$ (31)

$$\begin{array}{ccc} Q & = & \int Du\;\frac{\int_{-1}^{\infty }dW\;W^{2}\nu (u,W)}{\int_{-1}^{\infty }dW\nu (u,W)} \end{array}$$ (32)

$$\begin{array}{ccc} \Delta Q & = & \frac{1}{\sqrt{\hat{q}}}\int Du\;u\frac{\int_{-1}^{\infty }dW\;W\nu (u,W)}{\int_{-1}^{\infty }dW\nu (u,W)} \end{array}$$ (33)

$$\begin{array}{ccc} 0 & = & \int Du\;\frac{\int_{-1}^{\infty }dW\;W\nu (u,W)}{\int_{-1}^{\infty }dW\nu (u,W)} \end{array}$$ (34)

The integrals over *dW* in the last three equations can be performed explicitly, yielding:

$$\begin{array}{ccc} Q & = & \frac{\hat{q}+{\hat{M}}^{2}+\Delta \hat{Q}}{\Delta {\hat{Q}}^{2}}+\frac{1}{\Delta {\hat{Q}}^{\frac{3}{2}}}\int Du\;(u\sqrt{\hat{q}}-\hat{M}-\Delta \hat{Q})𝒢\;(-\frac{u\sqrt{\hat{q}}-\hat{M}+\Delta \hat{Q}}{\sqrt{\Delta \hat{Q}}}) \end{array}$$ (35)

$$\begin{array}{ccc} \Delta Q & = & \frac{1}{\Delta \hat{Q}}+\frac{1}{\sqrt{\Delta \hat{Q}\hat{q}}}\int Du\;uG(-\frac{u\sqrt{\hat{q}}-\hat{M}+\Delta \hat{Q}}{\sqrt{\Delta \hat{Q}}}) \end{array}$$ (36)

$$\begin{array}{ccc} 0 & = & -\frac{\hat{M}}{\Delta \hat{Q}}+\frac{1}{\sqrt{\Delta \hat{Q}}}\int Du\;G(-\frac{u\sqrt{\hat{q}}-\hat{M}+\Delta \hat{Q}}{\sqrt{\Delta \hat{Q}}}) \end{array}$$ (37)

#### Critical capacity

At critical capacity, the space of the solutions shrinks to a point, and the mutual overlap tends to become equal to the self overlap: *q* → *Q*, i.e. Δ*Q* → 0. In this limit, the conjugate order parameters diverge as:

$$\begin{array}{ccc} \hat{q} & = & \frac{C}{\Delta Q^{2}} \end{array}$$ (38)

$$\begin{array}{ccc} \Delta \hat{Q} & = & \frac{A}{\Delta Q} \end{array}$$ (39)

$$\begin{array}{ccc} \hat{M} & = & \frac{B\sqrt{C}}{\Delta Q} \end{array}$$ (40)

Using these conditions, and calling *α*_*c*_ the critical value of *α*, the saddle point equations, 29 to 34, become:

$$\begin{array}{ccc} Q & = & \frac{1}{A}(C-B\sqrt{C}) \end{array}$$ (41)

$$\begin{array}{ccc} A & = & H(B-\frac{A}{\sqrt{C}}) \end{array}$$ (42)

$$\begin{array}{ccc} 0 & = & \frac{\sqrt{C}}{A}(G(B-\frac{A}{\sqrt{C}})-BA)-(1-A) \end{array}$$ (43)

$$C\;=\;\alpha_{c}Q\;{\langle \left(1+\tau_{\sigma }^{2}\right)H\left(\tau_{\sigma }\right)-\tau_{\sigma }G\left(\tau_{\sigma }\right)\rangle }_{\sigma }$$ (44)

$$A\;=\;\alpha_{c}{\langle H\left(\tau_{\sigma }\right)\rangle }_{\sigma }$$ (45)

$$0\;=\;{\langle \sigma \left(G\left(\tau_{\sigma }\right)-\tau_{\sigma }H\left(\tau_{\sigma }\right)\right)\rangle }_{\sigma }$$ (46)

where we defined

$$\tau_{\sigma }=\frac{\sigma (M-T\sqrt{Q})-K}{(1-f)\sqrt{Q}}$$ (47)

These equations can be solved numerically to find the six parameters *α*_*c*_, *Q*, *A*, *B*, *C* and *M*.

Note that in the special case *K* = 0 these equations have a degenerate solution with *Q* = 0 and the same *α*_*c*_ as in the case of unbounded synaptic weights (e.g. *α*_*c*_ = 2 for *f* = 0.5). This is because in that case the original problem has the property that scaling all weights by a factor of *x* is equivalent to scaling the boundary $\bar{w}$ by a factor of *x* ^−1^ (see Eq 16); therefore, the optimal strategy is to exploit this property by setting *x* → 0, i.e. effectively reducing the problem to the unbounded case. Of course, this strategy can only be pursued up to the available precision in a practical setting.
